# Nutritional evaluation of soybean meals varying in particle size

**DOI:** 10.1016/j.psj.2023.102708

**Published:** 2023-04-14

**Authors:** E.M. Ahasic, P.L. Utterback, C.M. Parsons

**Affiliations:** Department of Animal Sciences, University of Illinois at Urbana-Champaign, Urbana, IL 61801, USA

**Keywords:** soybean meal, particle size, growth performance, amino acid digestibility, metabolizable energy

## Abstract

The objective of this study was to evaluate the effect of varying soybean meal (**SBM**) particle sizes on nutritional value of the SBM. Seven samples of dehulled solvent-extracted SBM from the same batch were ground to varying mean particles of <386, 466, 809, 1,174, 1,577, 2,026, and 2,321 μm. Two precision-fed rooster assays (crop intubation with 25 g of SBM followed by 48 h total excreta collection) were performed to determine TME_n_ and standardized amino acid (**AA**) digestibility. There were no significant differences for TME_n_ among SBM samples, and there was also no consistent significant effect of particle size on standardized AA digestibility. In addition to the 2 precision-fed rooster assays, a 21 d broiler chick trial was conducted using corn-SBM based diets using 4 diets that differed only in the mean particle size of SBM (466, 809, 1,174, or 1,577 μm), being fed from 2 to 23 d of age. Chicks fed diets containing 809 or 1,174 μm SBM had increased (*P* < 0.05) weight gain compared with chicks fed the diet containing 466 μm SBM, and chicks fed diets containing 1,174 or 1,577 μm SBM had increased (*P* < 0.05) feed efficiency compared with chicks fed the diet containing 466 μm SBM. The diet containing 466 μm SBM yielded the highest (*P* < 0.05) AME_n_ and total tract P retention. Ileal P digestibility and standardized AA digestibilities did not differ among treatments. Relative gizzard weight (percent of body weight) was increased (*P* < 0.05) by the 2 largest SBM particle sizes. The results from these 3 experiments showed that increasing SBM particle size may be beneficial to broiler growth performance and may increase gizzard size but had no consistent significant effect on ME, AA digestibility, or P digestibility/retention.

## INTRODUCTION

Feed costs are the largest expense to the poultry industry, and the 2 largest energy costs related to broiler feed manufacturing are pelleting and decreasing particle size, respectively (Reece et al., 1985; Amerah et al., 2007). Particle size has been a topic of interest in the poultry industry for a number of years, and increasing particle size of feed ingredients is a way to reduce feed manufacturing costs. Reece et al. (1986a) reported that increasing the screen size of a hammer mill from 4.76 to 6.35 mm increased mill capacity by 27% which could result in substantial energy savings. It has been reported that smaller particle sizes may positively affect growth performance and nutrient utilization because of the greater surface area made available to digestive enzymes (Amerah et al., 2007). However, numerous studies have reported that when ingredients with increased particle sizes are given to poultry, gizzard size is increased (Nir et al., 1994; Parsons et al., 2006; Pacheco et al., 2013). A larger gizzard may allow feed particles to remain for a longer period in the upper digestive tract, increasing the time that particles are exposed to digestive enzymes, resulting in increased digestion (Jones et al., 2001; Hetland et al., 2002).

Few studies have investigated the effect of soybean meal (**SBM**) particle size on growth performance and nutrient digestibility. Pacheco et al. (2013) observed increased body weights at 14, 35, and 49 d of age for chicks fed coarse (1,080 μm) versus fine (352 μm) expeller-extracted SBM but no difference in body weight at 35 and 49 d for chicks fed fine (465 μm) versus coarse (971 μm) solvent-extracted SBM. Also, protein digestibility was increased for larger particle size SBM (Pacheco et al., 2013). From 0 to 42 d, Marx et al. (2021) fed mash diets to broilers differing only in SBM particle size (625, 775, 1,053, or 1,406 μm), and observed that FCR linearly decreased as SBM particle size increased, but particle size did not influence feed intake. It was also observed that smaller or coarser particle size SBM resulted in similar CP digestibility and digestible energy (Marx et al., 2021). Thus, increasing the particle size of SBM may not only reduce energy needed during feed manufacturing but may also improve the growth performance and nutrient utilization of poultry. Because SBM is one of the main ingredients included in poultry diets and only a small number of studies have been conducted to evaluate SBM particle size, it is desirable to conduct further research (Pacheco et al., 2013; Marx et al., 2021; Lyons et al., 2023). The objectives of this study were to determine the TME_n_ and standardized amino acid (**AA**) digestibility of SBM varying in particle sizes utilizing the precision-fed rooster assay and to evaluate what effect SBM particle size may have on growth performance and nutrient digestibility in young broiler chicks.

## MATERIALS AND METHODS

The protocols for this study were reviewed and approved by the Institutional Animal Care and Use Committee (IACUC protocols 20131 and 22048).

### Ingredients and Analyses

A single sample of un-milled solvent-extracted SBM was acquired from a commercial solvent extraction facility located in the Midwest and processed to obtain 7 particle sizes at Texas A&M University, College Station, TX. The meal was sampled directly from the outlet of the desolventizer-toaster/dry-cooler; thus, it had not been exposed to any downstream milling or classification process.

The un-milled SBM was processed as follows. To facilitate minor crushing of the meal, a roller mill (shallow corrugations) (CPM Roskamp Champion, Waterloo, IA) was set up to both vary gap distance between the rolls and to reduce the roll speed, which was accomplished using a variable speed drive set to 30 Hz. A hammer mill (Bliss Eliminator, Ponca City, OK) was used in sequence if further particle size reduction was required. In a few instances, there were multiple passes through the roller mill as well as the need for a second pass through the hammer mill after switching to a screen with finer perforations in order to achieve the desired particle size. The mills were fed by hand.

A sieve shaker (Tyler Ro-Tap, Mentor, OH) was used to analyze in duplicate the particle size of each final fraction. The mean particle size values of the SBM were <386, 466, 809, 1,174, 1,577, 2,026, and 2,321 μm. The US Sieve stack was comprised of, from top to bottom, the following mesh numbers: 4, 6, 8, 12, 16, 20, 30, 40, 50, 70, 100, 140 pan.

Analyses were conducted on the SBM at a commercial laboratory (University of Missouri-Columbia Experiment Station Chemical Laboratory, Columbia, MO) to determine N for CP (Method 990.03; AOAC International, 2007), crude fat (Method 920.93 A; AOAC International, 2007), acid detergent fiber (**ADF**; Method 973.18; AOC International, 2007), neutral detergent fiber (**NDF**; Method 2002.04; AOAC International, 2007), and ash (Method 942.05; AOAC International, 2007). Mineral concentrations of Ca and P were determined using inductively coupled plasma optical emission spectroscopy (Method 985.01 A, B, and D; AOAC International, 2007). Concentrations of AA were analyzed (Method 982.30 E [a, b, and c]; AOAC International, 2007). Gross energy (**GE**) was analyzed using an adiabatic bomb calorimeter (Model 6300; Parr Instruments, Moline, IL) and DM content was determined (Method 930.15; AOAC International, 2007), at the University of Illinois at Urbana-Champaign.

### TME_n_ and Standardized Amino Acid Digestibility (Experiments 1 and 2)

Two precision-fed rooster assays were conducted to evaluate TME_n_ (Experiment 1) and AA digestibility (Experiment 2). In both experiments, single Comb White-Leghorn roosters were fasted for 26 h to empty their gastrointestinal tracts. After fasting, 6 roosters were tube-fed 25 g (crop intubation) of one 7 SBM samples. The mean SBM particle sizes fed in these experiments were < 386, 466, 809, 1,174, 1,577, 2,026, and 2,321 μm. Conventional roosters were used to determine TME_n_ and cecectomized roosters (Parsons, 1985) were used to determine standardized AA digestibility. After being tube fed, each rooster was placed in an individual wire cage over an excreta collection tray. Excreta (feces + urine) were quantitatively collected for 48 h after tube feeding. Excreta samples were freeze dried, weighed, and ground prior to being analyzed. Excreta samples from conventional roosters were analyzed for GE and N as described previously. Endogenous GE losses were determined using conventional roosters that were fasted for 48 h and TME_n_ was subsequently calculated as described by Parsons et al. (1982). The equation used to calculate TME_n_ is shown below:

$$\text{TM}E_{n}\;\text{of}\;\text{diets}\;\left(\text{kcal}/g\right)\;=$$

[GEconsumed−(GEexcretedbyfedbirds+8.22×Nretainedbyfedbirds)+(GEexcretedbyfastedbirds+8.22×Nretainedbyfastedbirds)]/feedintake(g)

where GE consumed (kcal) = diet intake (g) × GE of the diet (kcal/g); GE excreted by fed or fasted birds (kcal) = excreta output (g) × GE of excreta (kcal/g); 8.22 = GE (kcal) of uric acid per g of N (Hill and Anderson, 1958); N retained by fed or fasted birds (g) = diet intake (g) × diet N (%) − excreta output (g) × excreta N (%).

Excreta samples from the cecectomized roosters were analyzed for AA as described earlier. Endogenous AA losses were determined using cecectomized roosters that were fasted for 48 h, and standardized AA digestibility values were subsequently calculated using the method of Engster et al. (1985) using the equations below.

StandardizedAAdigestbilityofdiets(%)=[(AAconsumed−AAexcretedbyfedbirds+AAexcretedbyfastedbirds)/AAconsumed]×100

where AA consumed (g) = diet intake (g) × AA in diet (%); AA excreted by fed birds (g) = excreta output (g) × AA in excreta (%); AA excreted by fasted birds = excreta output (g) × AA in excreta (%).

### Broiler Chick Experiment (Experiment 3)

Four dietary treatments were used (Table 1). The diets were corn-SBM based with the only differing factor among diets being the particle size of the test SBM. Diets 1 to 4 contained 35.5% SBM of mean particle size 466, 809, 1,174, or 1,577 μm, respectively. Titanium dioxide was included in each diet at 0.40% to provide an indigestible marker. Diets were analyzed for DM, GE, N, P, and AA as described previously, and titanium was analyzed at the University of Missouri-Columbia Experiment Station Chemical Laboratory using the method of Myers et al. (2004). The experiment was a 21 d assay with diets fed from 2 to 23 d of age.

**Table 1:** Ingredient composition of experimental diets containing soybean meals varying in mean particle size in Experiment 3.

	Diet			
Ingredient, %	1	2	3	4
Corn	58.229	58.229	58.229	58.229
466 μm SBM^1^	35.488	–	–	–
809 μm SBM^1^	–	35.488	–	–
1,174 μm SBM^1^	–	–	35.488	–
1,577 μm SBM^1^	–	–	–	35.488
Soybean oil	2.049	2.049	2.049	2.049
Limestone	1.120	1.120	1.120	1.120
Dicalcium phosphate	1.250	1.250	1.250	1.250
NaCl	0.450	0.450	0.450	0.450
L-Lys HCl	0.167	0.167	0.167	0.167
DL-Met	0.300	0.300	0.300	0.300
L-Thr	0.082	0.082	0.082	0.082
Vitamin mix^2^	0.200	0.200	0.200	0.200
Trace mineral mix^3^	0.150	0.150	0.150	0.150
Choline Cl (60%)	0.100	0.100	0.100	0.100
Phytase^4^	0.015	0.015	0.015	0.015
Titanium dioxide	0.400	0.400	0.400	0.400
Analysis, %				
CP^5^	21.06	21.44	21.38	20.69
Met + Cys^5^	0.86	0.86	0.80	0.89
Lys^5^	1.34	1.28	1.27	1.32
Thr^5^	0.83	0.84	0.81	0.87
Ca^6^	0.81	0.81	0.81	0.81
Total P^5^	0.62	0.58	0.62	0.62
Na^6^	0.20	0.20	0.20	0.20

1 SBM: soybean meal.

2 Provided per kilogram of diet: retinyl acetate, 4,400 IU; cholecalciferol, 25 µg; DL-α-tocopheryl acetate, 11 IU; vitamin B_12_, 0.01 mg; riboflavin, 4.41 mg; D-pantothenic acid, 10 mg; niacin, 22 mg; and menadione sodium bisulfite complex, 2.33 mg.

3 Provided as milligrams per kilogram of diet: manganese, 75 from MnSO_4_·H_2_O; iron, 75 from FeSO_4_·H_2_O; zinc, 75 from ZnO; copper, 5 from CuSO_4_·5H_2_O; iodine, 75 from ethylene diamine dihydroiodide; selenium, 0.1 from Na_2_SeO_3_.

4 Optiphos 2000, Huvepharma, Sofia, Bulgaria. Supplied 300 FTU phytase/kg of feed.

5 Analyzed value.

6 Calculated value.

Ross 708 males were obtained from a commercial hatchery. Chicks were weighed, wingbanded, and allotted to pens at 2 d post hatch. Chicks were housed in Petersime batteries with raised wire floors in an environmentally controlled room. The experiment was a completely randomized design, with 4 dietary treatments, 10 replicate pens per treatment, and 5 chicks per pen for a total of 200 chicks. The average initial weight of the chicks was 47.2 g, with feed and water provided ad libitum. At the end of the 21 d experimental period, all chicks and feeders were weighed. Weight gain, feed consumption, and feed efficiency (gain:feed ratio) were calculated for each replicate pen. Chicks were euthanized on the last day of the experimental period via asphyxiation with carbon dioxide gas. Gizzards were then excised, separated from external fat and internal contents, rinsed, blotted dry, and subsequently weighed. To determine ileal P digestibility and AA digestibility, the section of the ileum posterior to Meckel's diverticulum and anterior to the ileal-cecal junction was excised from each bird. The ileal contents were collected by flushing with water combined by a small amount of gentle squeezing, and the digesta contents were pooled for the 5 chicks in each replicate pen. To determine AMEn, excreta samples were collected from pans under the pens from d 19 to 21 of the experimental period. Ileal and excreta samples were freeze dried and ground for subsequent analyses. Ileal and excreta samples were analyzed for titanium, P, and AA as described previously at the University of Missouri-Columbia Experiment Station Chemical Laboratory. Excreta samples were also analyzed for GE and N as described previously. The AME_n_ of the diets was calculated using the equations below as described by Hill and Anderson (1958) with titanium dioxide used as the indigestible marker.

$$\text{AM}E_{n}\;\text{of}\;\text{diets}\;\left(\text{kcal}/g\right)\;=\;\text{Ediet}\;-\;\text{Eexcrata}\;-\;8.22\;\times \;N\;\text{retained}$$

where Ediet = GE of diet (kcal/g); Eexcreta = GE in excreta (kcal/g) × [titanium in diet (%) / titanium in excreta (%)]; N retained (per g of diet) = N (per gram of diet) – [N (per g of excreta) × titanium in diet (%) / titanium in excreta (%)].

The apparent ileal AA digestibility (**AIAAD**) and standardized ileal AA digestibility (**SIAAD**) of the diets were calculated as follows:

AIAAD(%)=[(AAdiet−AAilealdigesta)/AAdiet]×100

where AAdiet = AA in the diet (%); AA ileal digesta = AA in ileal digesta (%) × titanium in diet (%) / titanium in ileal digesta (%)

The AIAAD values were then standardized using the basal ileal endogenous AA flow values (IEAA;mg/kg DM intake) for 21-day-old broiler chickens fed a N-free diet from the study of Adedokun et al. (2007b) and then the SIAAD values were calculated using the equations below as described by Adedokun et al. (2009):

SIAAD(%)=AIAAD(%)+[100×basalIEAAflow(g/kgDMintake)/AAindiet(g/kgDM)]

The equations described by Ravindran et al. (2005) were modified to calculate apparent values for ileal P digestibility and total tract P retention and are shown below:

IlealPdigestibility(%)=[(Pdiet−Pilealdigesta)/Pdiet]×100

Where Pdiet = P in the diet (%); P ileal digesta = P in ileal digesta (%) × titanium in diet (%) / titanium in ileal digesta (%)

TotaltractPretention(%)=[(Pdiet−Pexcreta)/Pdiet]×100

Where Pdiet = P in the diet (%); P excreta = P in excreta (%) × titanium in diet (%) / titanium in excreta (%)

### Statistical Analysis

Data from all 3 experiments were analyzed by ANOVA using the GLM procedure in SAS (SAS Institute, 2010). In addition, when there was a significant treatment effect of SBM particle size, the GLM procedure was used to conduct linear regression analyses to assess linear and quadratic effects of SBM particle size. In Experiments 1 and 2, each individually caged rooster served as the experimental unit. In Experiment 3, each pen of 5 chicks served as the experimental unit. The least significant difference test was used to assess differences among dietary treatments in all experiments. Significance was determined at *P* < 0.05 for all statistical comparisons. The least significance difference test was primarily used for discussing significant SBM particle size treatment effects since, with the exception of AMEn in Experiment 3, R^2^ values for the linear regression analyses were only 0.24 to 0.36 among the different measured parameters.

## RESULTS AND DISCUSSION

### Nutrient Analysis

The nutrient compositions among SBM samples (Table 2) were generally similar to one another, which was expected as the samples were all obtained from the same batch. The CP contents of the SBM samples were lower than the value of 48.5% reported by the NRC (1994) but are within the range of those reported by Ravindran et al. (2014) where SBMs from 4 different countries were found to have a mean CP content ranging from 46.9 to 48.2% and were also within the range of values reported for United States SBM samples in the meta-analysis study of Ibanez et al. (2020). Indeed, the mean CP of those samples in the latter study was 46.4% which is similar to the SBM herein. The analyzed DM and ADF contents from the SBM samples in the current study were also similar to the US SBM samples analyzed in the Ravindran et al. (2014) study, whereas the analyzed NDF values of the SBM in the current study were slightly lower than the NDF values for United States SBM in the Ibanez et al. (2020) study which ranged from 7.2% to 13.1% NDF with a mean of 8.69% NDF. The SBM samples from the current study had a slightly lower crude fat percentage than those from Ravindran et al. (2014) and Ibanez et al. (2020) and the NRC (1994) value of 1.0% fat. The SBM samples also had values for Ca and P consistent with those reported in the NRC (1994), which are 0.27% and 0.62%, respectively. The Ca level of the SBM in the current study (0.22%–0.29%) was, however, somewhat lower than the mean Ca level of 0.39% for the US SBM in the Ibanez et al. (2020) study.

**Table 2:** Analyzed composition and TME_n_ in Experiment 1 of soybean meals varying in mean particle size.

	<386 μm	466 μm	809 μm	1,174 μm	1,577 μm	2,026 μm	2,321 μm
DM (%)	89.4	88.9	88.4	88.2	87.7	87.5	87.5
CP (%)	46.6	46.3	46.4	46.2	47.1	46.9	46.4
Crude fat (%)	0.90	0.89	0.81	0.89	0.78	0.84	0.77
ADF (%)^1^	5.98	6.07	5.88	5.55	5.37	6.26	7.05
NDF (%)^1^	7.97	7.55	7.03	6.53	6.50	7.01	7.80
Ca (%)	0.28	0.29	0.28	0.28	0.28	0.27	0.27
Total P (%)	0.64	0.64	0.63	0.63	0.64	0.64	0.62
Ash (%)	6.07	6.04	6.02	5.94	6.02	5.95	6.04
TME_n_ (kcal/kg DM)^2^^,^^3^	2,863	2,871	2,857	2,844	2,880	2,797	2,910

1 ADF: acid detergent fiber; NDF: neutral detergent fiber.

2 TME_n_ values are means of 6 individually-caged conventional roosters.

3 Pooled SEM for TME_n_ is 53.6. There were no significant differences among means (*P* > 0.05).

The TME_n_ values (Table 2) did not differ significantly among SBM samples. The values for TME_n_ were slightly higher than the TME_n_ value of 2,761 kcal/kg DM reported in the NRC (1994) for dehulled solvent extracted SBM containing 1.0% fat. The TME_n_ values reported for the present SBM samples are similar to those determined by Parsons et al. (2000) who reported TME_n_ values of 2,835, 2,794, and 2,874 kcal/kg DM for 3 conventional SBM samples using conventional roosters and are also similar to the value of 2,775 kcal/kg DM for the TMEn of dehulled solvent extracted SBM reported by Chen et al. (2013).

The average total concentrations of selected AA among the SBM samples for Met, Lys, Thr, Val, Arg, and Ile were 0.65, 2.93, 1.78, 2.30, 3.31, and 2.21%, respectively (Table 3). Digestibility values did not differ significantly among SBM except for Cys, Arg, and Trp. For the 809 μm particle size, the digestibility value for Cys was lowest among SBM particle size samples and differed significantly (*P* < 0.05) when compared with the other 6 SBM particle sizes (Table 3). For Arg, digestibility for the 466 μm SBM was significantly higher than for the 809, 1,174, 1,577, 2,026, and 2,321 μm particle size SBM samples. In addition, the 809, 1,577, and 2,321 μm particle size SBM samples had significantly lower Arg digestibility when compared with the <386 and 466 μm particle size SBM. For Trp digestibility, the 466 μm particle size SBM had a significantly higher value than the 1,577, 2,026, and 2,321 μm particle size SBM. The 2,026 and 2,321 μm particle size SBM were significantly higher than the 1,577 μm particle size SBM sample for Trp digestibility. The 1,577 μm particle size SBM had the lowest Trp digestibility and differed significantly from the other 6 SBM particle sizes. Thus, overall, there was generally little or no consistent effect of SBM particle size on AA digestibility in roosters. In contrast to the in vivo TMEn and AA digestibility results in roosters, increased SBM particle size generally decreased in vitro digestibility of SBM (Lyu et al., 2021, 2022).

**Table 3:** Total amino acids, standardized amino acid digestibility values, and digestible amino acid concentrations (%) for soybean meals varying in mean particle size from the precision-fed rooster assay in Experiment 2.

	<386 μm			466 μm			809 μm			1,174 μm		
Amino acid	Total	Digest. value	Digest. conc.^1^	Total	Digest. value	Digest. conc.^1^	Total	Digest. value	Digest. conc.^1^	Total	Digest. value	Digest. conc.^1^
Indispensable												
Arg	3.25	93.1^ab^	3.03	3.27	94.1^a^	3.08	3.25	90.4^c^	2.94	3.34	91.9^bc^	3.07
Gly	1.97	–	–	1.94	–	–	1.93	–	–	1.98	–	–
His	1.21	89.0	1.08	1.19	90.7	1.08	1.18	88.4	1.04	1.21	90.9	1.10
Ile	2.27	89.2	2.03	2.13	90.2	1.92	2.14	87.2	1.87	2.21	89.8	1.98
Leu	3.53	88.6	3.13	3.52	90.3	3.18	3.50	86.9	3.04	3.58	89.7	3.21
Lys	2.95	87.0	2.57	2.91	88.9	2.59	2.88	85.7	2.47	2.96	88.1	2.61
Met	0.65	88.2	0.57	0.64	89.5	0.57	0.63	86.3	0.54	0.66	88.5	0.58
Phe	2.36	89.8	2.12	2.38	91.3	2.17	2.38	88.4	2.10	2.43	90.7	2.20
Thr	1.74	85.2	1.48	1.79	87.2	1.56	1.76	83.5	1.47	1.80	86.5	1.56
Trp	0.68	95.8^ab^	0.65	0.64	96.4^a^	0.62	0.68	95.3^ab^	0.65	0.67	95.9^ab^	0.64
Val	2.37	85.5	2.03	2.22	86.3	1.92	2.23	82.7	1.84	2.31	85.9	1.98
Dispensable												
Ala	2.01	84.1	1.69	1.99	86.5	1.72	1.98	82.3	1.63	2.03	85.6	1.74
Asp	5.15	89.0	4.58	5.15	90.1	4.64	5.10	87.8	4.48	5.24	89.6	4.70
Cys	0.69	78.7^a^	0.54	0.67	81.1^a^	0.54	0.66	72.6^b^	0.48	0.68	80.0^a^	0.54
Glu	8.05	91.3	7.35	8.06	92.9	7.48	8.03	90.8	7.29	8.24	92.6	7.63
Pro	2.37	89.2	2.11	2.38	90.6	2.16	2.42	86.9	2.10	2.45	89.5	2.19
Ser	1.78	86.9	1.55	1.97	89.4	1.76	1.90	85.8	1.63	1.93	88.2	1.70
Tyr	1.49	89.9	1.34	1.61	91.6	1.48	1.59	88.5	1.41	1.62	90.6	1.47

	1,577 μm			2,026 μm			2,321 μm					
Amino acid	Total	Digest. value	Digest. conc.^1^	Total	Digest. value	Digest. conc.^1^	Total	Digest. value	Digest. conc.^1^	Pooled SEM^2^		
Indispensable												
Arg	3.36	90.5^c^	3.04	3.35	91.9^bc^	3.08	3.32	91.1^c^	3.03	0.68		
Gly	1.98	–	–	1.99	–	–	1.96	–	–	–		
His	1.23	89.5	1.10	1.23	90.2	1.11	1.22	89.9	1.10	0.65		
Ile	2.24	88.9	1.99	2.27	89.3	2.03	2.22	88.9	1.97	0.91		
Leu	3.61	89.0	3.21	3.59	88.8	3.19	3.56	88.5	3.15	0.97		
Lys	2.97	86.5	2.57	2.98	86.8	2.59	2.96	87.2	2.58	0.92		
Met	0.66	87.5	0.58	0.66	88.1	0.58	0.66	88.5	0.58	1.06		
Phe	2.45	90.1	2.21	2.44	90.1	2.20	2.41	89.9	2.17	0.80		
Thr	1.83	86.0	1.57	1.79	85.9	1.54	1.76	84.9	1.49	1.06		
Trp	0.47	93.0^c^	0.44	0.57	94.8^b^	0.54	0.67	95.0^b^	0.64	0.45		
Val	2.34	85.5	2.00	2.34	85.3	2.00	2.33	84.6	1.97	1.10		
Dispensable												
Ala	2.04	84.5	1.72	2.04	84.0	1.71	2.01	84.5	1.70	1.31		
Asp	5.32	88.8	4.72	5.27	89.4	4.71	5.18	88.7	4.59	0.68		
Cys	0.69	78.5^a^	0.54	0.69	79.0^a^	0.54	0.68	79.6^a^	0.54	1.67		
Glu	8.24	91.8	7.56	8.25	92.2	7.61	8.10	91.7	7.42	0.57		
Pro	2.44	88.7	2.16	2.32	87.9	2.04	2.42	88.9	2.15	0.76		
Ser	1.90	88.2	1.68	1.90	87.9	1.67	1.85	87.2	1.61	1.04		
Tyr	1.62	89.9	1.46	1.57	89.1	1.40	1.61	89.8	1.44	0.80		

a-c Standardized digestibility values within the same row with no common superscripts differ (P < 0.05). Values are means of 6 individually caged cecectomized roosters.

1 Digestible concentration = (total × standardized digestibility values)/100.

2 Pooled SEM for standardized digestibility values.

Standardized AA digestibility values determined in the present study for most AA were similar to those determined in SBM using the precision-fed rooster assay by Kim et al. (2012). Because there was no consistent effect of SBM particle size on AA digestibility values among the 7 varying SBM particle sizes, a larger particle size SBM could possibly be used in poultry diets to help reduce feed costs and increase output of the milling process. Reece et al. (1986a) reported a 37% increase in grinding output from a hammer mill when the screen size was increased from 3.18 to 4.76 mm to grind corn.

The effect of particle size on pelleting or pellet quality of the mixed feed is also a topic of interest. For studies conducted with corn, Reece et al. (1986b) observed that fineness of grain had no effect on pellet durability. Also, Chewning et al. (2012) reported no significant differences for feed efficiency between birds fed a pelleted diet including 300 μm corn and those fed a pelleted diet including 600 μm corn at 14, 21, 35, or 44 d of age. However, Rubio et al. (2020) reported that particle size did influence pellet quality in finisher diets. They found that diets containing 2,613 μm corn had a higher pellet durability index than diets containing 615 μm corn. It was found, though, that corn particle sizes greater than 1,644 μm may negatively impact FCR during the finisher phase. However, corn particle size did not influence BW, feed intake, or FCR during the grower and starter phases when birds were fed crumbled diets, and particle size did not influence pellet quality of the grower diets (Rubio et al., 2020). These studies with corn suggest that increased particle size in a pelleted diet may not greatly impact growth performance. For SBM, Lyons et al. (2023) reported that finely ground SBM (hammer mill fitted with a 2.4 mm screen) yielded improved pellet quality for diets compared with diets containing SBM ground through 5.6 or 7.9 mm screens. Additional research, however, needs to be conducted to determine what effect SBM particle size may have on pellet quality and durability, as well as any impact SBM particle size may have on broiler performance when SBM is included in pelleted diets.

In Experiment 3, the analyzed concentrations of CP, AA, and minerals in the 4 diets were generally similar (Table 1) and any observed variation among diets was probably due to sampling and/or analytical variation. Results for weight gain, feed intake, feed efficiency, AME_n_, ileal P digestibility, and total tract P retention of chicks fed diets differing in SBM particle size are shown in Table 4. Chicks fed the diet containing 1,174 μm SBM gained the most weight, which was significantly different than the weight gain observed from feeding the diets containing 466 or 1,577 μm SBM. These results are not in total agreement with those reported by Pacheco et al. (2013). In that study, chicks fed 1,080 μm expeller-extracted SBM had greater BW than chicks fed 352 μm expeller-extracted SBM at 14, 35, and 49 d age. In addition, chicks fed a diet containing 971 μm conventional SBM gained less weight when compared with chicks fed a diet containing 465 μm SBM at 14 d; however, this difference was not present at 35 or 49 d. (Pacheco et al., 2013). It is possible that the diet containing 1,577 μm SBM particle size of the current study may have been too large for the chicks, particularly at the youngest ages, to utilize optimally, resulting in the significantly lower weight gain compared with chicks fed the diet containing 1,174 μm SBM. Similar conclusions were made in other studies where it was concluded that large particle size SBM (>1300 μm) may have a negative impact on growth performance in very young chicks (Pacheco et al., 2013; Marx et al., 2021). In the current study, feed consumption did not differ for chicks fed diets containing 466 to 1,174 μm SBM. Feed consumption for chicks fed the diet containing 1,577 μm SBM, however, was significantly lower than for chicks fed the other diets, which again could be attributed to the particles being too large for the chicks to consume. Marx et al. (2021) came to a similar conclusion when it was observed that 1,406 μm SBM resulted in the lowest feed consumption for 21-day-old broilers compared with diets containing 625, 775, or 1,053 μm SBM. In the present study, chicks fed the diets containing 1,174 or 1,577 μm SBM had higher feed efficiency than chicks fed the diets containing the smaller particle size SBM. The latter difference for the diet containing 1,577 SBM may be at least partially due to the fact that the chicks from this treatment consumed the least amount of feed, possibly resulting in decreased feed wastage and decreased fat deposition. Similarly, Marx et al. (2021) found that FCR decreased linearly as SBM particle size increased in diets fed to broilers from d 0 to 48. However, from 0 to 21 d, SBM particle size had a quadratic effect on BWG and FCR, with the diets containing 775 or 1,053 μm SBM resulting in increased BWG and improved FCR compared with the diets containing 625 or 1,406 μm SBM. Also, from 0 to 21 d, the researchers observed a linear decrease in feed consumption as SBM particle size increased (Marx et al. 2021). Another possible reason for the increased feed efficiency for the larger particle size SBM observed in the present study is that the breakdown of larger feed particles in the upper intestine may be slower than finer particles, resulting in increased peristalsis, which in turn may result in increased utilization of feed (Nir et al., 1995). In contrast to the growth performance effects discussed above for broiler chicken studies, Lyons et al. (2022) did not observe any impact of SBM particle size on pullet performance from 0 to 17 wk of age.

**Table 4:** Growth performance of broiler chicks in Experiment 3.^1^

SBM^2^ particle size (μm)^3^	Weight gain (g/chick)	Feed intake (g/chick)	Gain:feed (g/kg)	AME_n_ (kcal/kg DM^2^)	Ileal P digestibility (%)	Total tract P retention (%)
466	840^c^	1,241^a^	680^c^	3,243^a^	58.1	54.4^a^
809	901^ab^	1,272^a^	709^bc^	3,047^b^	58.4	48.8^b^
1,174	912^a^	1,257^a^	726^ab^	2,973^c^	55.4	49.9^b^
1,577	875^bc^	1,156^b^	761^a^	2,961^c^	55.4	50.5^b^
Pooled SEM	12.3	26.5	14.2	20.0	1.36	0.89

a-c Means within a column with no common superscript differ (*P* < 0.05). Significant linear effect of SBM particle size for gain:feed and a significant quadratic effect for weight gain, feed intake, AME_n_, and total tract P retention.

1 Values are means of 10 pens of 5 chicks from 2 to 23 d of age for body weight gain, feed intake, and gain:feed ratio. The AME_n_, ileal P digestibility, and total tract P retention values are at 23 d of age for 10 pens of 5 chicks.

2 DM: dry matter; SBM: soybean meal.

3 Diets differed only in the particle size of the SBM included. Diets are differentiated by the particle size of the included SBM.

When comparing the current results for SBM to those obtained in a study with corn, Downs et al. (2023) observed no significant treatment effects for BW or feed conversion among broilers fed diets containing 832, 1,432, or 2,036 μm corn. It was observed, however, that FCR worsened with increasing particle size (Downs et al. 2023). The latter results and the results of the current study suggest that particle size for corn may have larger effects on broiler growth performance than SBM particle size because there were no significant differences between the diets containing 809 and 1,174 μm SBM for growth performance in the current study. The results from the present study indicate that increasing SBM particle size to 1,174 μm may be beneficial in improving broiler growth performance; however, a 1,577 μm SBM particle size may be too large for young chicks for maximum growth and feed consumption.

The AME_n_ values for the diets generally decreased as SBM particle size increased (significant quadratic effect; R^2^ = 0.76). The AME_n_ value for the diet containing 466 μm SBM was significantly higher than for the other diets which contained larger particle size SBM. The AME_n_ value for the diet containing 809 μm SBM was also significantly higher than the diets containing 1,174 or 1,577 μm SBM. These results were not consistent with the rooster assay which showed no significant differences in TME_n_ among the SBM varying in mean particle size from <386 to 2,321 μm. The reason for the differences in particle size effects between roosters and broiler chickens is unknown. At first glance, it could be hypothesized that differences were due to bird age. It has been shown that nutrient digestibility and ME increase with increasing age in broiler chickens (Noy and Sklan, 1995; Batal and Parsons, 2002), and endogenous amino acid losses decrease with increasing age (Adedokun et al., 2007a); however, these age effects were primarily observed only in birds between 0 and 10 to 15 d of age. The AME_n_ values for broiler chickens in the current study were determined at 23 d of age. Thus, there may be factors other than age that are responsible for the lack of SBM particle size on TMEn in roosters compared with AME_n_ in broiler chickens. For other studies with broiler chickens, Kilburn and Edwards (2004) observed no significant differences for AME_n_ values when feeding broilers corn-SBM diets containing either 891 or 1,239 μm SBM. For 42-day-old broilers, Marx et al. (2021) found that ileal digestible energy was increased for diets containing 625 or 1,406 μm SBM and decreased for diets containing 775 or 1,053 μm SBM. Jacobs et al. (2010) observed a linear decrease in diet AME_n_ as corn particle size increased at 7 d of age in broilers. Those diets contained mean corn particle sizes of 557, 858, 1,210, or 1,387 μm. This result, however, was not observed at 21 d. The researchers attributed the 7 d results to the very young broilers fed the coarse particle size corn having gizzards not developed enough to sufficiently grind the particles, resulting in decreased early growth performance (Jacobs et al., 2010). The latter hypothesis may apply to the present study because the SBMs varying in particle size were included in the diets starting at 2 d of age. However, the negative effect of increasing SBM particle size on AME_n_ values in the current study was contradictory to the improved feed efficiency response with increasing SBM particle size. The reason for the disagreement in effect of SBM particle size on AME_n_ versus feed efficiency is unknown. As mentioned earlier, perhaps the larger particle SBM resulted in less feed wastage and subsequent increased feed efficiency, with this positive response on measured feed efficiency being larger than any negative effect of reduced AME_n_.

Ileal P digestibility did not differ significantly among treatments. Total tract P retention, however, was increased significantly in the diet containing 466 μm SBM when compared with the diets containing larger particle size SBM. Total tract P retention did not differ among the diets containing 809, 1,174, or 1,577 μm SBM. Kilburn and Edwards (2004) found no significant difference in P retention when feeding broilers corn-SBM diets containing 891 or 1,239 μm SBM; however, tibia ash was increased with increased SBM particle size. Mtei et al. (2019) also did not observe any differences in apparent ileal P digestibility when broilers were fed corn-SBM diets containing 492, 700, or 912 μm corn. It is possible that the P in smaller particle size SBM in the current study was more effectively utilized than in the larger particle SBM contained in the other diets, resulting in increased total tract P retention; however, this result is not consistent with the growth performance and ileal P digestibility data.

Gizzard weights and relative gizzard weights (% of body weight) increased as SBM particle size increased (Table 5). When comparing gizzard weights, diets containing 1,174 or 1,577 μm SBM resulted in the largest gizzards that were significantly greater than those of chicks fed the diet containing 466 μm SBM. Gizzard weight of chicks fed the diet containing 809 μm SBM was not significantly different from those of chicks fed any of the other diets. The diet containing 1,577 μm SBM resulted in the significantly largest relative gizzard weight (% of BW), and relative gizzard weight of chicks fed the diet containing 1,174 μm SBM was also significantly higher than for chicks fed the diet containing 809 μm SBM. Many studies have observed larger gizzard weights with increased particle sizes for many different feed ingredients (Parsons et al., 2006; Amerah et al., 2008; Pacheco et al., 2013). One study with SBM, however, found that neither gizzard weight nor yield were influenced by SBM particle size (Lyons et al., 2023). That study, however, only evaluated SBM particle sizes of approximately 571, 603, and 670 μm, whereas the present study evaluated a wider range of particle sizes. Additionally, Lyons et al. (2023) attributed the lack of SBM particle size effect possibly to the reduction of the particle size of feed ingredients during conditioning and pelleting. The larger gizzard weights observed with increased particle size SBM could be at least partially responsible for the improved growth performance observed when SBM particle size increased in the current study. A more developed gizzard may stimulate gastric function and digestion by increasing the secretion of cholecystokinin, which stimulates the gastro-duodenal refluxes and secretion of pancreatic enzymes, increasing the time and extent to which the digesta are exposed to an acidic environment (Duke, 1992; Li and Owyang, 1993; Svihus et al., 2004; Mtei et al., 2019). A lower pH of gizzard contents may also increase pepsin secretion, resulting in increased protein digestion (Gabriel et al., 2003). Also, coarse particles may slow the rate of passage of digesta through the gastrointestinal tract, increasing the time that digesta are exposed to digestive enzymes (Nir et al., 1994).

**Table 5:** Gizzard weights of chicks in Experiment 3.^1^

SBM^2^ particle size^3^ (μm)	Gizzard weight (g)	Gizzard weight (% of body weight)
466	14.63^b^	1.65^bc^
809	15.52^ab^	1.64^c^
1,174	16.26^a^	1.70^b^
1,577	16.23^a^	1.77^a^
Pooled SEM	0.31	0.02

a-c Means within a column with no common superscript differ (*P* < 0.05). Significant linear effect of SBM particle size for both gizzard weight (g) and gizzard weight (% of body weight).

1 Values are means of 10 pens of 5 chicks at 23 d of age.

2 SBM: soybean meal.

3 Diets differed only in the particle size of the SBM included. Diets are differentiated by the particle size of the included SBM.

Amino acid digestibility did not differ significantly for any AA among dietary treatments (Table 6), and this lack of SBM particle size effect in broiler chickens is consistent with the rooster results in Experiment 2. Ganzer et al. (2007) reported that diets containing 1,010 μm SBM resulted in decreased CP and AA digestibility for 3-wk-old broilers when compared with 630 μm SBM (Ganzer et al., 2007). Additionally, Siegert et al. (2018) observed that reducing hammermill grid size from 3 to 2 mm, thus reducing the proportion of coarse particles, significantly increased pre-cecal AA digestibility in pelleted diets fed to commercial broilers from age 14-21 d. Lyu et al. (2021) observed that in vitro CP digestibility decreased with increasing SBM particle size during the range of 57 to 1000 μm. In contrast, Pacheco et al. (2013) observed increased protein digestibility with increasing mean SBM particle size to 1,290 from 470 μm for expeller-extracted SBM. Also, Lyons et al. (2023) found that diets containing approximately 571 μm SBM resulted in the lowest digestibility for all indispensable AA in 21-day-old broilers and found that feeding diets containing the coarsest particle size tested (approximately 670 μm) resulted in improved Thr, Met, and Lys digestibility compared with a diet containing the finest (approximately 571 μm) particle size tested. Interestingly, for older 42-day-old broilers, Marx et al. (2021) observed that CP digestibility of SBM was similar for diets containing 625 μm or 1,406 μm particle size SBM; however, diets containing 775 μm or 1,053 μm SBM resulted in decreased CP digestibility when compared with diets containing the 665 or 1,406 μm SBM. Thus, the effect of particle size on digestibility of CP and AA in SBM for broiler chickens is inconsistent and unclear at present.

**Table 6:** Standardized amino acid digestibility values for broilers fed corn-soybean meal diets containing soybean meals varying in particle size in Experiment 3.^1^

	Corn-SBM Diet^2^				
Amino acid	466 μm	809 μm	1,174 μm	1,577 μm	Pooled SEM
Indispensable					
Arg	90.7	90.4	88.9	90.3	0.72
Gly	81.4	80.9	78.2	80.3	0.99
His	87.2	86.8	84.7	86.3	0.76
Ile	85.2	84.9	82.9	84.7	0.91
Leu	86.1	85.8	83.9	85.5	0.87
Lys	87.0	87.1	84.6	87.0	1.04
Met	93.5	93.5	92.0	93.2	0.62
Phe	86.3	85.9	84.2	85.6	0.83
Thr	80.6	80.4	77.6	79.8	1.03
Trp	86.1	85.9	83.8	86.0	0.85
Val	81.9	81.8	79.2	81.1	0.97
Dispensable					
Ala	85.6	85.4	83.3	85.1	0.91
Asp	83.6	83.1	81.1	82.7	0.80
Cys	75.9	75.0	71.4	74.5	1.20
Glu	89.7	89.4	87.9	89.2	0.65
Pro	85.4	84.6	82.8	84.8	0.69
Ser	83.3	83.2	80.0	82.3	0.91
Tyr	85.6	85.2	83.3	84.9	0.85

1 Values are means of 10 pens of 5 chicks at 23 d of age. There were no significant effects of soybean meal particle size for any amino acid (*P* > 0.05).

2 Diets differed only in the particle size of the SBM included. Diets are differentiated by the particle size of the included SBM.

In conclusion, the results of the current study indicate that increasing the particle size of SBM, with 1,174 μm being a possible approximate optimal particle size, may improve broiler growth performance and increase gizzard size, with the latter having potential benefits for increasing nutrient digestibility. No consistent effect, however, was observed for SBM particle size on ME, AA digestibility or P digestibility/retention.
